# Illuminating Brain Activities with Fluorescent Protein-Based Biosensors

**DOI:** 10.3390/chemosensors5040032

**Published:** 2017-11-28

**Authors:** Zhijie Chen, Tan M. Truong, Hui-wang Ai

**Affiliations:** 1 California Institute for Quantitative Biosciences, QB3, University of California, Berkeley, CA 94720, USA; 2 Center for Membrane and Cell Physiology, and Biomedical Sciences (BIMS) Graduate Program, University of Virginia, Charlottesville, VA 22908, USA; 3 Department of Molecular Physiology and Biological Physics, University of Virginia, Charlottesville, VA 22908, USA; 4 Department of Chemistry, University of Virginia, Charlottesville, VA 22908, USA

**Keywords:** fluorescent proteins, biosensors, fluorescent probes/indicators, brain imaging, genetically encoded calcium indicators, genetically encoded voltage sensors, genetically encoded glutamate sensors, synaptic activity indicators, pH indicators, bioluminescent sensors

## Abstract

Fluorescent protein-based biosensors are indispensable molecular tools for life science research. The invention and development of high-fidelity biosensors for a particular molecule or molecular event often catalyze important scientific breakthroughs. Understanding the structural and functional organization of brain activities remain a subject for which optical sensors are in desperate need and of growing interest. Here, we review genetically encoded fluorescent sensors for imaging neuronal activities with a focus on the design principles and optimizations of various sensors. New bioluminescent sensors useful for deep-tissue imaging are also discussed. By highlighting the protein engineering efforts and experimental applications of these sensors, we can consequently analyze factors influencing their performance. Finally, we remark on how future developments can fill technological gaps and lead to new discoveries.

## 1. Introduction

The human brain has an average of 86 billion neurons, forming complex neuronal networks that are essential for behavior, intelligence, learning, and memory [1]. A fundamental goal in neuroscience has been to dissect neuronal circuits in vivo and to elucidate how identified populations of neurons contribute to behavioral outputs [2]. In fact, understanding brain function is not only a challenging scientific quest aiming to disentangle the functional relationship between the electrical, chemical, and hemodynamics (the dynamics of blood flow) in the brain with the cognitive, emotional, and behavioral outputs [3], but it’s also a clinically crucial endeavor toward the study, diagnostics, treatment, and intervention of neurological diseases, such as Alzheimer’s [4], stroke [5], and epilepsy [6]. As brains are structurally compact and functionally susceptible to invasive manipulations, optical imaging is highly compelling for interrogating their function [7].

The past 30 years have seen rapid development of brain imaging techniques [8]. Information obtained through brain imaging facilitates both functional interpretation and medical advancements toward addressing neurological diseases. While each method provides unique merits in studying brain activities, it also accompanies certain pitfalls that prevent one technique to dominate. For example, imaging modalities, such as functional magnetic resonance imaging (fMRI) [9], positron emission tomography [10], and X-ray computed tomography (X-ray CT) [11] have established themselves as clinically important tools for neurosurgical planning and diagnosing [12]. However, these techniques can only provide limited spatiotemporal information about brain function. Complementary to these techniques are electrophysiology and optical neuroimaging, through which electric and neurochemical signals in the brain can be detected and further related to neuronal and cortical functions [13]. While electrophysiology, the ‘gold standard’ for investigating neuronal functions [14, 15], can directly measure electrical activity of cells with high sensitivity, the invasive requirement of physical contact with tissues and the accompanied poor spatial resolution lend its dominant position in neuroscience to be challenged by the emerging optical neuroimaging techniques [16]. The use of light to study neuronal signaling has several advantages, including limited invasiveness, tunable wavelength, high spatial resolution, and sensitive detection [17].

**Figure 1:**
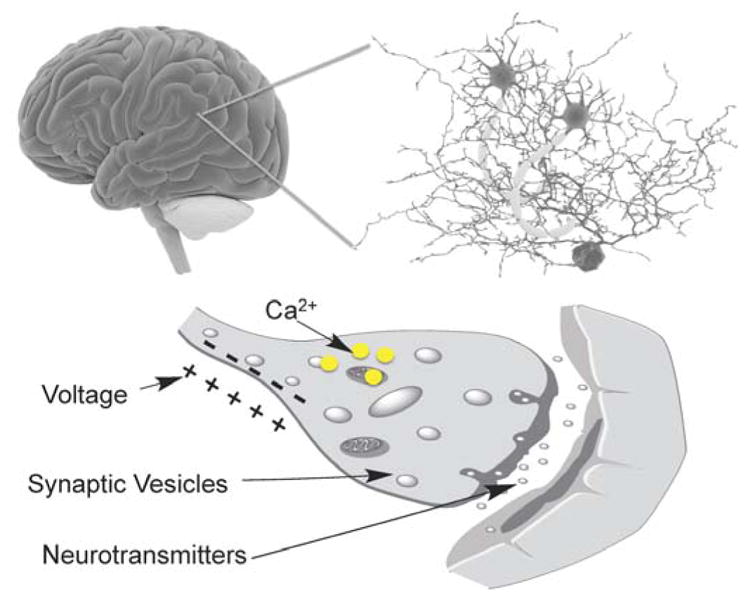
Biochemical events associated with neuronal activity The brain contains a large and complex neuronal network that consists of billions of neurons and astrocytes. Biochemical signals that have been successfully probed with genetically encoded fluorescent probes include membrane voltage (electric activity), intracellular Ca^2+^, synaptic vesicle recycling, and neurotransmitter release.

Optical neuroimaging measures neuronal activities by converting neuronal signals, such as voltage, calcium ions (Ca^2+^), and neurotransmitters into light signal outputs (Figure 1). It is thus an indirect approach towards studying neuronal signaling that necessitates the use of reporters (or probes, sensors, indicators, etc.) [16]. As such, the invention of optical reporters for brain imaging has been a highly intensive area of research [18]. Historically, calcium- [19] and voltage-sensitive dyes [20] have played pivotal roles in optically recording brain signals in vitro and in vivo. The use of dyes, however, is invasive and may result in non-specific staining. Moreover, the spatial and temporal localization of dyes cannot be easily controlled, making targeted or chronic imaging difficult. As a complementary and competing technology, genetically encoded fluorescent indicators are becoming increasingly popular because they (1) can be introduced into cells through transgenic DNA expression; (2) enable targeted expression with precise spatial and temporal control; (3) facilitate large-scale recording of neuronal activities in vitro and in vivo; (4) are amenable to protein engineering and optimizations; (5) are relatively noninvasive; and (6) are suitable for chronic imaging. However, since genetically encoded fluorescent indicators often require genetic delivery, they are less appropriate for direct, in vivo applications on human subjects. The past two decades has witnessed the fast development of a growing list of genetically encoded fluorescence probes, which have found broad applications within the neuroscience community for analyzing neural circuit functions [21–23]. With an increasing number of probes being successfully applied for circuit interrogation in vivo, it is now evident that they are transforming neuroscience in an unprecedented manner [21]. While reviews for Ca^2+^ and voltage sensors are available, very few of them covers genetically encoded indicators for various phenotypic effects of neuronal transmission and evaluate them within a unified framework [24]. To this end, we summarize the history and recent development in the field of optical sensors for neuronal activities, with a focus on indicators for Ca^2+^, voltage, neural transmitters, and pH changes.

## 2. Genetically Encoded Ca^2+^ Indicators (GECI)

Indicators for intracellular Ca^2+^ dynamics are widely used tools in neuroimaging. Following an action potential, Ca^2+^ levels show a transient, sharp, and localized increase within the cytosol, which is characteristic of promoting signal propagation at axon terminals [25, 26]. Thus, detecting intracellular Ca^2+^ fluctuations is a proxy for probing neuronal action potentials. Similar Ca^2+^ transients also occur in astrocytes, which play an essential role in synaptic plasticity [25, 26]. Typically, Ca^2+^ transients rise within a few milliseconds and last for hundreds of milliseconds, which are manifested as Ca^2+^ spikes [27]. GECIs have a rich history of both design and application (Table 1), having been so well optimized that many serve as the default indicator for preliminary investigations into neuronal signaling [28]. As GECIs have been subjected to extensive protein engineering; their large dynamic range and optimal response kinetics can facilitate large-scale recordings of neuronal activities in vivo [29, 30].

### 2.1. GECIs Based on FRET

Soon after the crystallization and high resolution structural determination of *Aequorea victoria* GFP [65], genetically encoded fluorescent probes based on Fluorescence (Förster) Resonance Energy Transfer (FRET) emerged as attractive alternatives to organic fluorophores for studying intracellular signaling molecules, most notably with Ca^2+^ [66] and cAMP [67]. In 1997, the Tsein lab reported the first proof-of-concept GECI, called cameleon-1 [31], which consists of a tandem fusion of BFP, *Xenopus* calmodulin (CaM) [68–70], CaM-binding peptide of myosin light-chain kinase-M13 [71], and GFP. Ca^2+^ binding to cameleon-1 triggers a conformational switch of CaM-M13 from a dumbell-like, extended form to a compact, globular form, causing a decrease in the distance and/or altering of the dipole orientation of the flanking FP in order to increase the FRET efficiency. Different signal peptides fused to cameleon-1 allows researchers to visualize free, localized Ca^2+^ dynamics in the cytosol, nucleus, and ER in HeLa cells [31]. Subsequent iterative optimizations can re-purpose and improve characteristic features of these GECIs. To improve the overall dynamic range and reduce pH sensitivity of cameleons, Miyawaki et al. developed a series of Yellow Cameleons (YCs) by replacing the original BFP–GFP pair with an ECFP–EYFP pair, resulting in a YC2.1 variant with a dynamic response of 100%, which was used to image Ca^2+^ in hippocampal neurons [32].

The structural modularity of FRET-based GECIs has facilitated further independent attempts toward improving YCs as an efficient indicator, such as via substitution of donor and acceptor FPs with enhanced photophysical properties as they became available, reorganization of the CaM-M13 sensing module, evolution of the CaM-Ca^2+^ binding site, and modulation of the linker length and/or composition between CaM-M13 and the flanking FPs [35]. For example, substitution of EYFP in YC2.1 with Citrine (YC2.3 and YC3.3) [45] or Venus (YC2.12) [72] FP produced GECIs with improved protein folding and maturation at 37 °C, that are indifferent to chloride ions, and that are more resistant to pH fluctuations at physiological conditions. Insertion of CaM binding peptide of CaM-dependent kinase kinase (CKKp) [73] into the linker region that connects the two EF-hands of CaM resulted in YC6.1, a GECI that increased the dynamic range of YC2.1 to reach 200% [34]. Despite the aforementioned improvements, these GECIs still suffer from a poor signal-to-noise ratio (SNR), especially when targeted to organelles or submicroscopic environments. To engineer high SNR, FRET-based GECIs, Nagai and co-workers employed a circularly permuted YFP (cpYFP) as the FRET acceptor [33]. Since cpYFPs have new N- and C-termini in close proximity to the chromophore [44], their use as FRET acceptors would result in different FRET performances, compared to their wild-type counterparts, due to an alteration of the relative orientation and dipole of the donor and acceptor chromophores. Accordingly, after sampling circularly permutated Venus (cpVenus) at different permutation sites and substituting them in YCs, they found that cp173Venus absorbs a greater amount of energy from excited CFP donor, thereby producing YC3.6 with Ca^2+^-dependent FRET ratio change of nearly 600%. Its large dynamic range enabled imaging of stimulated, fast-Ca^2+^ dynamics of hippocampal brain slices from YC3.6-genetically encoded transgenic mice [33]. YC3.6 has since become one of the most frequently used GECIs.

**Figure 2:**
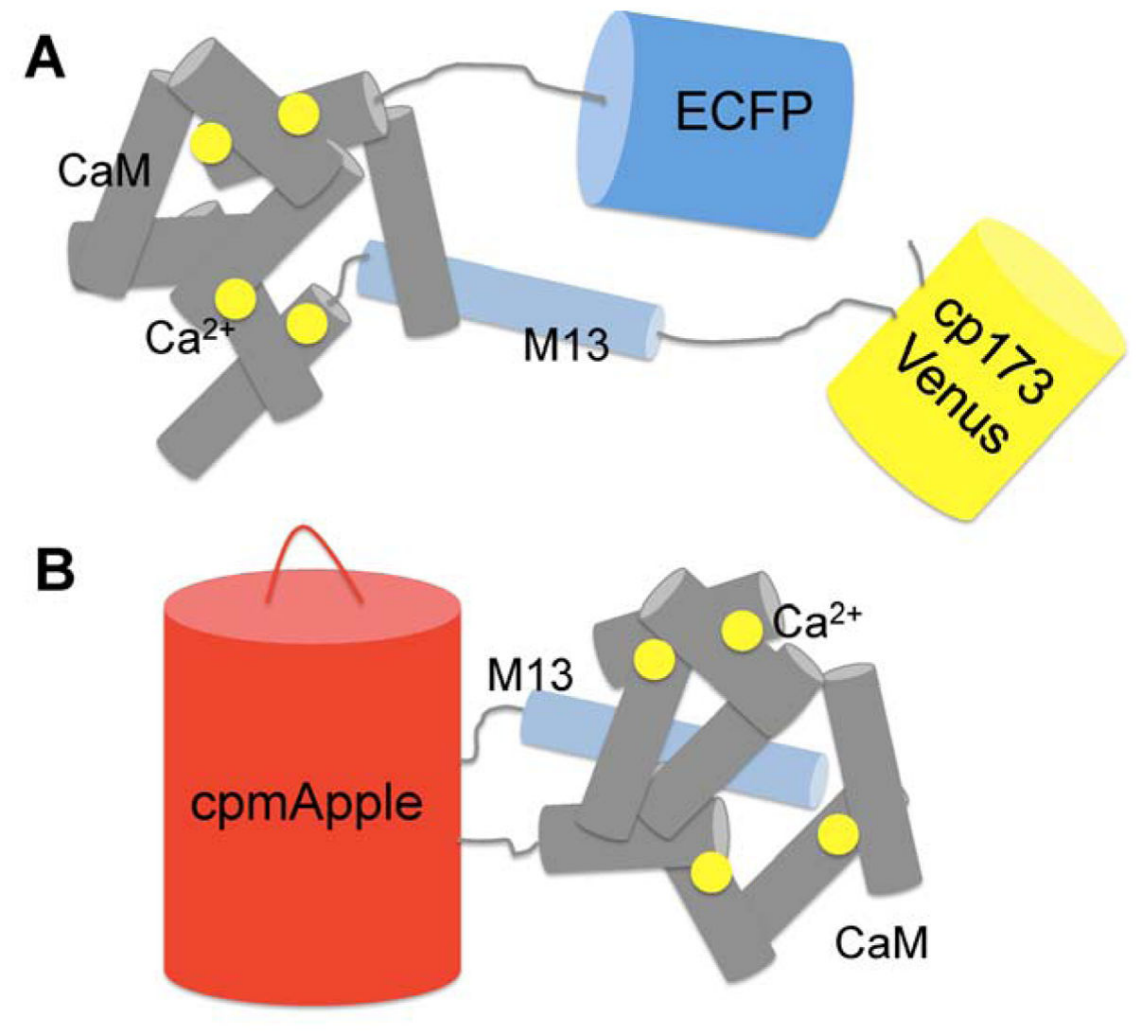
Schematic representation of genetically encoded Ca^2+^ indicators (**A**) YC-Nano is a FRET-based Ca^2+^ Indicator. CaM and M13 are sandwiched between ECFP and cp173Venus. Upon Ca^2+^ binding, conformational change induces an increase in FRET efficiency; (**B**) R-GECO1 is a red fluorescent GCaMP type Ca^2+^ Indicator based on a single red fluorescent protein. It consists of cpmApple, M13 fused to the N-terminal and CaM fused to the C-terminal. Upon Ca^2+^ binding, the conformational change of the CaM-M13 complex leads to local chromophore environment change, accompanying a large increase of the red fluorescence intensity of the sensor.

Of equal importance to an indicator’s dynamic range is its Ca^2+^ binding affinity, which dictates GECI sensitivity in a given biological context [74]. To this end, Palmer et al. engineered improved cameleons (D2cpV, D3cpV, and D4cpV) based on computationally redesigned calmodulin-peptide pairs with Ca^2+^ sensitivities tuned to more than a 100-fold range (0.6–160 μM) [35]. In 2010, Horikawa and colleagues developed ultrasensitive GECIs, termed yellow cameleon-Nano (YC-Nano, K_d_ = 15–140 nM), by adjusting the length of the linker peptide between CaM and M13 (Figure 2A) [36]. Together with its large signal change (14.5-fold) in the presence of Ca^2+^, YC-Nano enabled imaging of spontaneous motor activities in living zebrafish embryos [36]. To date, YC-Nano remains popular for detecting subtle Ca^2+^ transients and basal-level neuronal activity in multicellular networks. For example, transgenic mice expressing YC-Nano15 or YC-Nano50 allowed ultrasensitive imaging of exocytotic events associated intracellular Ca^2+^ dynamics of pancreatic acinar cells [75], and subtle, transient, and local activity in the fine processes of astrocytes (Ca^2+^ twinkle) [76], respectively.

An apparent drawback to these YC-type GECIs resides is their use of CaM and M13 peptide as the Ca^2+^ sensing module, which can potentially interfere with cellular biochemical machineries [77]. CaM is a ubiquitous and tightly regulated signal protein that interacts with a multitude of CaM-binding proteins [78] to activate and modulate kinases, phosphatases, and ion channels [79]. Therefore, expression of CaM-containing GECIs may inadvertently perturb normal cellular functions to an unpredictable extent. Moreover, unwanted binding of M13 to endogenous CaM can compromise sensor functions. Indeed, earlier attempts of using cameleons in transgenic invertebrates have encountered various problems, such as attenuated indicator performance in vivo and suboptimal targeting efficiencies [80–82]. To create minimally perturbing, and more “bio-orthogonal” GECIs, Griesbeck and colleagues explored the use of troponin C (TnC) [83], a skeletal and cardiac muscle-specific Ca^2+^-binding protein [84, 85], to construct new types of GECIs. Inserting truncated or full-length TnC variants between CFP and Citrine leads to several TN-type GECIs, among which TN-L15 shows a 140% dynamic response and was targeted to the plasma membrane of HEK293 cells and primary hippocampal neurons [37]. Further engineering of the Mg^2+^- and Ca^2+^-binding sites within the C-terminal lobe of TnC, and use of alternative donor/acceptor pair (ECFP/Citrine cp174), improved the ion selectivity, dynamic range, and response kinetics of TN-L15, resulting in TN-XL with a 400% ratiometric change upon Ca^2+^ binding. With a fast off-rate and large dynamic range, TN-XL enables fast, stable, and reproducible imaging of presynaptic motor neuron terminals of transgenic fruit flies [38]. Subsequently, domain rearrangement (i.e., deleting the lower-affinity N-terminal and concatenating the remaining C-terminal lobe of TnC) and mutagenesis inside EF hands III and IV, gave rise to a more sensitive GECI, designated TN-XXL [39], which can be used for chronic, in vivo, two-photon imaging of mouse visual cortex. By replacing the ECFP/cpCitrine pair with a 3xCFP/cpVenus pair, Liu et al. further improved the dynamic range of TN-XXL by nearly 11-fold [40]. More recently, the Griesbeck lab reported another TnC-based GECI, called “Twitch,” which was developed by using the minimized C-terminal domain of *Opsanus tau* troponin C (tsTnC), which has fewer Ca^2+^-binding sites, higher Ca^2+^-binding affinity, and lower potential buffering of endogenously Ca^2+^ [41]. Interestingly, the authors optimized the FRET responses of the Twitch indicators with a two-step, large functional screen: first in bacterial colonies and then in rat hippocampal neuronal cultures. The performance of the resulting indicators, such as Twitch-3 and Twitch-2B, rivals that of the synthetic dye, Oregon Green BAPTA1-AM [86, 87]. They demonstrated the usefulness of the Twitch indicators by recording visually evoked calcium signals in mouse cortical layer II/III neurons and imaging T lymphocyte activation [41].

### 2.2. GECIs Based on BRET

While FRET-based GECIs have well distinguished themselves as powerful tools for neuronal imaging, the idea of deploying BRET (bioluminescent resonance energy transfer) for GECI design was a rare but viable one. In 2010, Saito et al. reported an auto-luminescent Ca^2+^ sensor, BRAC [42], which is comprised of a Ca^2+^-sensing module (CaM-M13) sandwiched between a fluorescent protein acceptor (Venus) and a luciferase donor (RLuc8) [88, 89]. Ca^2+^ binding induced a conformational change within CaM-M13, followed by a 60% change in BRET efficiency [42]. They subsequently improved the BRET efficiency of the indicator and developed Nano-lantern (Ca^2+^ indicator) by inserting CaM-M13 in a N-terminally truncated R-Luc8 of an enhanced Nano-lantern BRET pair. Nano-lantern has a 300% BRET dynamic range and was successfully used in concert with channelrhodopsin2 (ChR2) [90–92] in dissociated rat hippocampal neurons to conduct optogenetic activation and functional imaging experiments. By swapping the donor in existing FRET- or BRET-based biosensors with a recently developed, highly bright luciferase, NanoLuc [93], new chemiluminescent BRET biosensors were derived. CalfluxVTN is a genetically encoded NanoLuc based-chemiluminescent Ca^2+^ biosensor modified from Twitch3 that can be used to couple optogenetic stimulation with Ca^2+^ sensing [94]. Moreover, enhanced Nano-lanterns (eNLs) and an eNL-based Ca^2+^ sensor with a 500% dynamic range have been recently reported [95]. BRET-based GECIs have the advantage of excitation-free, lower phototoxicity, and deeper tissue imaging capacity, but suffer from low photon output and the need of delivering bioluminescent substrates to points of interest. The recent development of a brighter and red-shifted teLuc luciferase [96] and the progress in engineering high efficiency BRET pairs in general is expected to partially alleviate this problem [43, 94–96].

### 2.3. Single FP-Based GECIs

Historically, GECIs based on single-FPs flourish, having more popularity than FRET-based GECIs. Although different in many aspects of their working principles and photophysical properties, GECIs based on single-FPs and FRET share common grounds in their developmental history, from proof-of-principle ideas, to reiterative optimizations, to universal adoptions. In 1999, Tsien and colleagues reported an interesting finding, showing that major structural rearrangements of GFP are tolerable, including circular permutation and domain insertion [44]. This finding paved the foundation for designing new biosensors using circularly permutated fluorescent proteins (cpFPs), affording the first single-FP based GECI, termed “Camgaroo1”, by insertion of CaM at Y145 of EYFP [44]. EYFP substitution for Citrine in camgaroo1 with Citrine lead to camgaroo2, which has improved expression at 37 °C, higher photostability, and reduced sensitivity to pH and chloride ions [45]. Despite proof-of-concept demonstrations, camgaroo-type GECIs failed to prove practical for Ca^2+^ imaging in live cells, primarily due to their unfavorable Ca^2+^ binding affinities (apparent K_d_ for Ca^2+^ is ~7 μM). However, the subsequent adoption of cpFPs [44, 97] in GECI designs led to indicators with significantly better performances. In 2001, two Japanese groups independently reported single-FP based GECIs, designated G-CaMP [47] and pericam [46], which are comprised of CaM and M13 fused to the N- and C-termini of a cpFP, respectively. These GECIs not only exhibit large dynamic ranges, but also have Ca^2+^ affinities (0.2–2 μM) within physiological ranges (50–5000 nM) [26], and are capable of imaging Ca^2+^ dynamics in subcellular compartments and mouse myotubes [46, 47]. Incorporation of known beneficial mutations for GFP gave rise to several G-CaMPs, including G-CaMP1.6 [48] and G-CaMP2 [49], with improved folding and maturation but marginal change to the dynamic range. Transgenic organisms expressing G-CaMP1.6 or G-CaMP2 were used to monitor neuronal activities of presynaptic boutons of the *Drosophila* larval neuromuscular junction [48], and mice cerebellar parallel fibers [49], respectively. In both cases, however, significant attenuation of SNR in vivo was observed [48, 49], highlighting the need for improved G-CaMPs. In a study of cell polarization during *Xenopus* gastrulation, Shindo et al. briefly mentioned GCaMP4.1 but did not detail how it was developed [51]. Muto et al. tested substitution of “superfolder GFP” [98] mutations into cpEGFP of GCaMP2 and developed GCaMP-HS (GCaMP-hyper sensitive) [52], which is capable of imaging spatiotemporal activation of the spinal motor neurons in zebrafish. Attempts to further optimize these single-FP based GECIs, not surprisingly, proved difficult, owing to the intricacies and unpredictability of CaM-M13 triggered environment changes around the cpFP chromophore. To aid rational design of improved G-CaMPs, two research groups independently determined the X-ray crystal structure of G-CaMP2 and elucidated its Ca^2+^-dependent fluorescence transformation mechanism: Ca^2+^ binding to CaM stabilizes a deprotonated chromophore in its otherwise protonated unbound state [50, 99]. Soon afterwards, Tian and coworkers used a combination of G-CaMP2 structure-guided mutagenesis and semi-rational library screening to develop G-CaMP3 [50], a new G-CaMP with increased baseline fluorescence, increased dynamic range, and higher affinity for Ca^2+^. GCaMP3 outperforms all previous GECIs, including GCaMP2, D3cpVenus, and TN-XXL in pyramidal cell dendrites, and was successfully used to image neuronal activities in worms, flies, and mice [50]. As a state-of-art GECI at that time, GCaMP3 soon become the default indicator for various imaging applications, including imaging neuronal activity in the motor cortex [50], barrel cortex [100], and hippocampus [101] of mice; chronic imaging of learning-related circuit changes in mice in vivo [102]; imaging light-evoked responses in neuronal populations of zebrafish tectum [103]; *Drosophila* [104] and mouse retina [105]; and probing dendritic excitation in mouse cortical layer V dendrites in vivo [106]. Meanwhile, further improvements of GCaMP3 continued. Through structure-guided, targeted mutagenesis, linker modulation, and high-throughput screening, Looger’s laboratory created a family of “GCaMP5” sensors with a wide range of Ca^2+^ affinities, dynamic range, baseline fluorescence, and on-off kinetics [53]. GCaMP5s have been systematically characterized in cultured neurons and astrocytes, and in animal models, including *Caenorhabditis elegans*, *Drosophila*, zebrafish, and mice, where single action potential (AP)-evoked signals are clearly detectable [53].

While the brightness, Ca^2+^ affinity, and dynamic ranges of GCaMPs are approaching those of synthetic Ca^2+^ indicator dyes, such as OGB1-AM (Oregon Green Bapta-1-AM) [106, 107], their sensitivities and kinetics are still a far cry. GCaMPs typically have half time for rise (τ_on_) and decay (τ_off_, the time from peak fluorescence to 50% of the peak fluorescence was defined as the half time decay) of τ_on_ = 20 ms–1.4 s and τ_off_ = 0.4–5 s, respectively, which is much slower than physiological Ca^2+^ flux signaling (rise within 1 ms and fall within 10–100 ms) [108], disfavoring their use to resolve spike times and firing rate variations over synthetic dyes (τ_on_ <1 ms and τ_off_ = 7 ms for OGB-1) [54]. Almost concomitantly, two groups reported their attempts, wherein they focused mutagenesis on CaM and the cpGFP-CaM interface to address this issue. Sun et al. developed Fast-GCaMPs, which shows up to a 20-fold accelerated off-responses and K_d_ values spanning from 0.16 to 6 μM, and can track natural song in *Drosophila* auditory neurons as well as the rapid responses in mammalian neurons [54]. Chen et al. reported a family of ultrasensitive protein calcium sensors (GCaMP6) and showed that GCaMP6 is the first GECI to outperform OGB-1 in vivo [55]. Notably, these GCaMP6 sensors (GCaMP6s, 6m, and 6f for slow, medium, and fast kinetics, respectively) were directly screened from GCaMP variants expressed in dissociated rat hippocampal neurons, underscoring the importance of direct sensor screening in physiologically relevant settings. To date, GCaMP6 remains one of the most advanced GECIs for use in vivo.

Just as GCaMP indicators had been gaining satisfactory acceptance, Campbell’s research group enthusiastically stepped into another untouched territory, engineering GCaMP spectral variants. Despite a decade of availability, the hue of single FP-based GECIs has, until 2011, been limited to monochromatic green. A red version of GCaMP indicator would be of particular interest in that it enables deeper tissue imaging, reduced phototoxicity, multicolor Ca^2+^ imaging with other GCaMP indicators, and multiparameter imaging with existing indicators derived from other species. In a landmark study in 2011, Zhao et al. developed a blue, improved green, and red ratiometric excitation and emission Ca^2+^ indicators with large dynamic ranges (Figure 2B) [56]. These indicators were developed from a high-throughput, colony-based screening system in which indicator variants are secreted to the periplasmic space of *E. coli*. Manipulation of Ca^2+^ concentrations in the periplasm by spraying Ca^2+^ or EGTA solutions to LB agar plates altered the Ca^2+^ on–off state, and thus the fluorescence of the indicators, which was then quantified by an imaging system [56]. To distinguish this new palette of indicators from the prior GCaMP series, a new term was coined for them, GECO (genetically encoded calcium indicators for optimal imaging). Multicolor imaging of Ca^2+^ was successfully achieved in HeLa cells transfected with nucleus-localized R-GECO1, cytoplasmic G-GECO1, and mitochondria-localized GEM-GECO1 [56]. In addition, they performed multiparameter imaging of Ca^2+^ and ATP by using R-GECO1 and ATeam1.03 [109]. Subsequently improved GECOs, such as R-CaMP1.07 [58] and R-GECO1.2 [57], GECOs with Ca^2+^ affinities suitable for mitochondria and endoplasmic reticulum (i.e., LAR-GECO1 [62]), and GECOs of other spectral variants, such as O-GECO1 [57], CAR-GECO1 [57], and Y-GECO1 [59], have since followed. More recently, a GECO with a large Stokes shift with suitability for two-photon imaging [110], namely REX-GECO1, was developed and used in organotypic hippocampal slice cultures and the visual system of albino tadpoles [60]. GR-GECOs are green-to-red photo-convertible GECOs that can be used as an optical “highlighter” for Ca^2+^ within a population of specified cells [61]. This diverse lineup of GECOs should find broad applications in neuronal imaging [22, 111]. Independently, Akerboom et al. rationally designed and engineered G-CaMP color variants, of which the blue (BCaMP1c), cyan (CyCaMP1a), and yellow (YCaMP1h) indicators were developed by grafting GFP mutations that produced blue [112], cyan [113] and yellow [65] FPs, whereas the red (RCaMP1h) indicator was developed by using a cp version of mRuby [114] within the GCaMP scaffold [63]. Interestingly, RCaMP1h, when used in combination with optogenetic actuators [90–92, 115–118], such as blue light-activatable channelrhodopsin-2 (ChR2) [90–92], was found to be superior than R-GECO1, which undergoes reversible photoactivation upon blue and green light illumination [63]. Coupling optogenetic manipulation with functional Ca^2+^ imaging using the ChR2/RCaMP1h pair enables the investigation of a signal input-output relationship within defined neuronal populations, both intra- and inter-cellularly (such as with neurons and astrocytes) [63].

Advancements in FP technology itself have also inspired new GECI designs. A recent example of a green–red ratiometric Ca^2+^ indicator based on FP exchange (FPX) technology underlies such new possibilities [64]. The tripartite, single-polypeptide Ca^2+^ biosensor, with the structure RA-CaM-B-M13-GA, employs dimerization-dependent green [119] and red [119] FPs (GA, RA), which share a common dimerization partner (B). Ca^2+^ binding shifts the equilibrium between RA-B and GA-B, resulting in a ratiometric red-to-green signal change [64]. The apparent advantage of FPX technology lies in its simple configuration from which new sensors can be quickly derived before extensive optimizations. The shortcomings, however, are that sensors derived from FPX technology are not suitable for quantitative imaging [64].

## 3. Genetically Encoded Voltage Indicators (GEVIs)

While tracking Ca^2+^ dynamics represents a robust way for detecting downstream signaling events following membrane depolarization, transmembrane voltage measurements provide more direct information on neural activity [120]. During the course of an action potential, the membrane potential undergoes a rapid change that results in a single signal spike or subthreshold oscillation [121]. Transmembrane voltage fluctuations can lead to subsequent downstream Ca^2+^ signaling or synaptic transmission within a single neuron, and the underlying information can also be relayed across long distances. Moreover, subthreshold membrane potential fluctuations cannot be detected by GECIs because they do not lead to any change in Ca^2+^ concentrations. Therefore, voltage indicators are intrinsically attractive tools for observing neuronal activities [122]. However, the lack of well-established voltage-sensing platforms together with the relatively faster temporal voltage fluctuations makes the design and application of voltage indicators far more daunting compared to Ca^2+^ indicators. Nevertheless, the progress in the development of engineering genetically encoded voltage indicators (GEVIs) has been exciting (Table 2) [123], and as we shall see, many voltage indicators of various classes have already found practical utility in vivo [124, 125]. Several important aspects of GEVIs including sensor development, sensing mechanism, and in vivo applications are also reviewed elsewhere [24, 123, 126–129].

### 3.1. GEVIs Based on Voltage Sensitive Domains (VSDs)

Naturally found in some ion channels or phosphatases, voltage sensitive domains (VSDs) are membrane-bound components that bear voltage-sensing capabilities [158, 159]. Typically, they contain four structural transmembrane helices (S1–S4) that modulate the gate of ion channels or control phosphatase activities. A defining feature that has been repeatedly harnessed by GEVI engineers is that the positively charged fourth helix (S4), or certain loop regions of VSDs, undergo substantial conformational changes upon membrane hyperpolarization or depolarization [123, 160]. Not surprisingly, coupling the conformational change of VSDs to fluorescence perturbations, either in the form of intensity change of a single FP or FRET efficiency change between two FPs, has been the principle guiding strategy in early GEVI designs. In 1997, Siegel et al. reported the first GEVI, FlaSh, which is composed of GFP inserted into a non-conducting mutant of Shaker K^+^ channel, and demonstrated that FlaSh GFP fluorescence can faithfully report membrane potential changes in *Xenopus laevis* oocytes [130]. Several improved GEVIs have since followed [136]. GFP replacement with other FP variants and/or rational incorporation of mutations known to alter gating kinetics and voltage dependence of the Shaker K^+^ channel, yielded FlaSh variants with improved folding and distinct spectra, kinetics, and voltage dependence [161]. Use of a reversibly non-conducting form of the rat μI skeletal muscle voltage-gated sodium channel led to SPARC, which sports a faster response kinetics [131]. Tandem fusion of CFP-YFP to the C-terminal of S4 of a K_V_ potassium channel produced a FRET-type GEVI, VSFP1 [132]. Yet, none of these first generation GEVIs gained widespread utility, predominately due to their poor mammalian expression and membrane trafficking [162].

**Figure 3:**
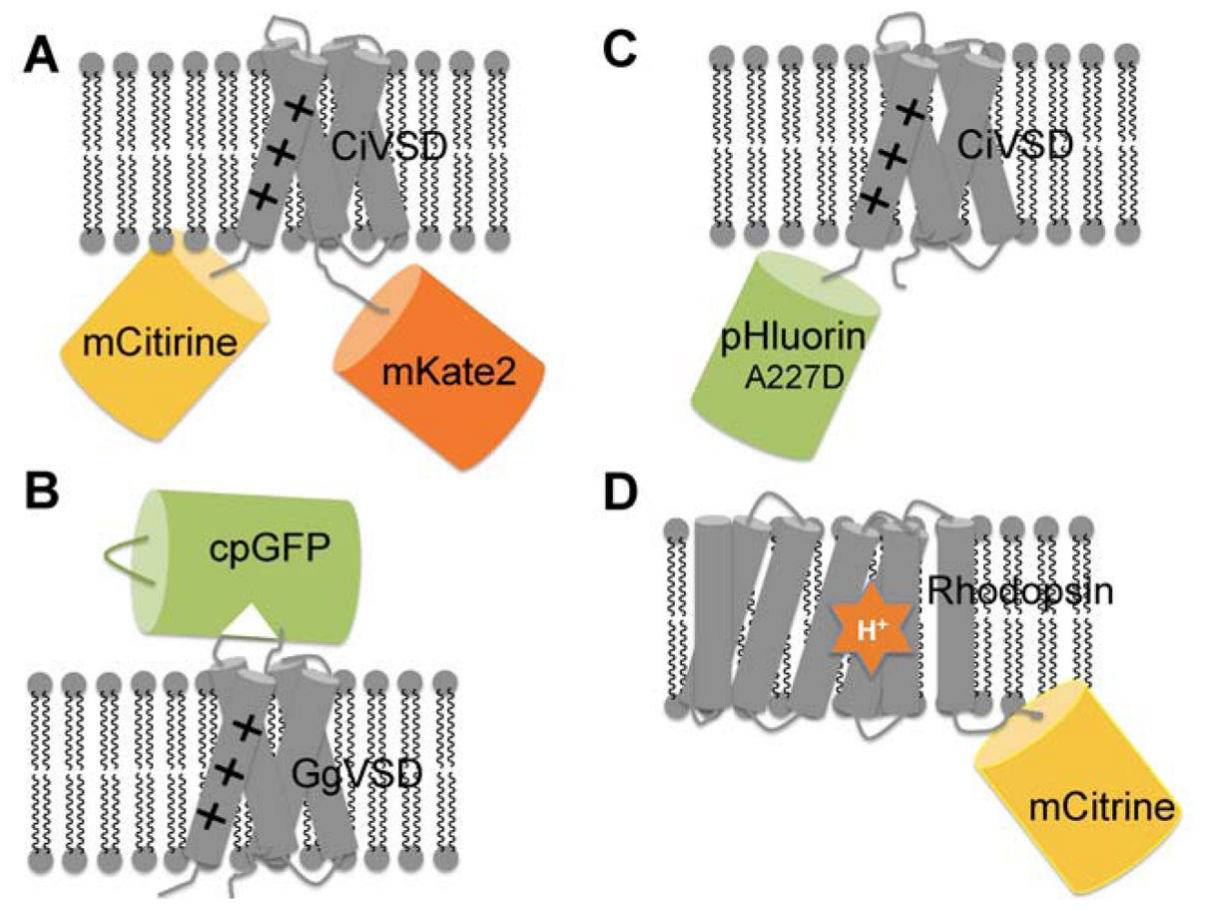
Schematic representation of genetically encoded voltage indicators (**A**) VSFP-butterfly1.2 is a FRET-based voltage sensor consists of mCtirine, CiVSD and mKate. Membrane depolarization induces a conformational change of CiVSD, which alters the FRET efficiency between the donor and acceptor; (**B**) ASAP1 is a single FP based voltage sensor made by inserting cpGFP in the S3–S4 linker of GgVSD. The conformational change of GgVSD can be transduced to cpGFP, leading to voltage dependent green fluorescence change; (**C**) ArcLight consists of Ci-VSD and super ecliptic pHluorin (A227D). Compared to other FP, this mutant FP has a higher sensitivity to the conformational change of CiVSD; (**D**) MacQ-mCitrine is a hybrid voltage reporter consisting a rhodopsin (MacQ) as FRET acceptor and a yellow fluorescent protein (mCitrine) as FRET donor. Membrane depolarization protonates the Schiff base of the bound retinal (orange star) cofactor in rhodopsin, shifting the cofactor absorption spectrum and enhancing FRET efficiency from mCitrine to the weakly fluorescent retinal cofactor.

This membrane-targeting problem [163] was largely solved in the second generation GEVIs by using the VSD from the voltage-sensitive phosphatase of the sea squirt, *Ciona intestinalis* (CiVSD) [164]. CiVSD has both sequence and functional homology to the VSD of Kv channels, and it exhibits very fast gating currents (~1 ms) in mammalian cells; but unlike the multimeric Kv channels, it can exist as an exclusive monomer [134]. It was postulated that deployment of CiVSD in these GEVIs reduced co-assemble with native channel subunits, and thus increased membrane localization capability and response kinetics [165, 166]. The first of such CiVSD-based GEVIs is VSFP2.1, which preserves the overall FRET configuration of VSFP1 but displays clear membrane localization and response to physiological neuronal membrane signals in PC12 cells [133]. And much like FRET-based GECIs, the modular design of VSFP2.1 (CiVSD-mCerulean-mCitrine) provided room for further improvements. For example, truncation of the five-amino-acids linker between CiVSD and mCerulean-mCitrine of VSFP2.1 resulted in VSFP2.3 [134], which has larger response amplitude. Replacement of mCerulean-mCitrine with alternative FRET pairs, such as mCitrine-mKate2 [167], mUKG-mKOκ [141], Clover-mRuby2 [137] led to VSFP2.42 [135], Mermaid [141], and VSFP-CR [137], respectively. Meanwhile, Knöpfel’s laboratory also pioneered the design of VSFP-butterfly [139] which have their FRET pairs inserted between the CiVSD instead of normally being tandemly fused. In cultured neurons, VSFP-butterfly showed reliable membrane targeting, high sensitivity to subthreshold electrical activity, fast kinetics for single-cell synaptic responses, and a high SNR [139]. VSFP-butterfly derivatives, including VSFP-butterfly1.2 (Figure 3A) [139] and Mermaid2 [142], are also among the first GEVIs to be used in vivo. Despite this progress, VSFP2s still suffered from slow repolarization kinetics. To address this issue, Mishina et al. developed chimeric VSDs (Ci-VSP-Kv3.1 VSD chimeras) by transplanting homologous motifs from the tetrameric voltage-activated potassium channel, Kv3.1, to the monomeric CiVSD [136]. Applying these chimeric VSDs to VSFP2.3 and VSFP-Butterfly1.2 led to the CiVSD-Kv3.1 chimera (C5) [136], and VSFP-butterfly CY(YR) [140] or Mermaid2 [142], respectively. Fortunately, these GEVIs maintained the dynamic range of their parental constructs while having much faster repolarization kinetics (13.4 ms for CiVSD-Kv3.1 chimera (C5) compared to 91.6 ms for VSFP2.3) [136].

Several attempts to fuse a single FP directly to the C-terminus of CiVSD only yielded GEVIs [138] with small dynamic ranges (<3%) such as VSFP3.1 [134] and VSFP3.1_mOrange2 [138]). A rare and exceptional example is ArcLight (Figure 3C) [143], a GEVI with a surprisingly large dynamic response (18.1%) over a physiological voltage range (−70 mV to +30 mV). The large response amplitude of ArcLight and of its linker variants, such as ArcLight A242 and ArcLight Q239 (~35%) [143], are believed to have originated from an unintended point mutation A227D within the super ecliptic pHluorin [168], although the detailed mechanism is still insufficiently understood [169]. In cultured hippocampal neurons, ArcLight variants allow reliable detection of single APs and subthreshold electrical events [143]. More recently, Cao et al. demonstrated that ArcLight enables precise optical measurements of membrane potentials from intact neuronal circuits of whole *Drosophila* brain [170], presaging a bright future of in vivo optical electrophysiology with GEVIs. Combinational mutagenesis of CiVSD, or swapping of CiVSD with VSDs from other species in ArcLight, gave rise to GECIs with faster kinetics but attenuated response amplitudes, such as in Bongwoori [145] and chicken ArcLight [144], respectively. Direct evolution of key residues in the FP domain of ArcLight produced Marina, a GEVI that exhibits a ΔF/ΔV with a positive slope relationship [147].

One shared caveat to the abovementioned GEVIs is their relatively slow response kinetics (typically >10 ms) when considering the time-scale of action potentials (~1 ms [171]), thereby compromising the ability of these GEVIs to effectively detect subthreshold potentials and rapid trains of APs. To address this issue, the Lin group recently developed a fast and highly responsive probe (2 ms on-off kinetics, 18–29% ΔF/F), ASAP1 (Figure 3B) [148], whereby they inserted a cpGFP into the S3–S4 loop of chicken VSD (GgVSD). Efficient transduction of VSD conformational changes to the chromophore environment of cpGFP is thought to be responsible for the accelerated kinetics, as was also similarly observed in VSD-cpGFP tandem fusion constructs [172, 173]. Owing to its fast kinetics and large dynamic range, ASAP1 faithfully detected single APs and subthreshold potential changes at kHz frame rates [148]. Recently, improved variants of ASAP1 with higher sensitivity and more rapid kinetics including ASAP2s [149] and ASAP2f [150] has been developed and applied with two-photon imaging, revealing unique insights into neural processing in subcellular domains and neuronal tissues. Using a similar strategy, Abdelfattah and co-workers developed a red shifted GEVI (FlicR1) that can be used in conjunction with a blue-shifted channelrhodopsin for all-optical electrophysiology [146].

### 3.2. GEVIs Based on Microbial Rhodopsins

Another emerging class of GEVIs, whose development was pioneered by Cohen and coworkers, leverages the use of microbial rhodopsins as both the voltage sensing and reporting element [174]. Rhodopsins are typical membrane-bound G-protein coupled receptors (GPCRs) that function as channels, ion pumps, or light sensors [91, 175]. Mechanistic studies of microbial- and bacterio-rhodopsins identified retinal and a retinal-lysine Schiff-base linkage in the protein core as key components in the photocycle that confers light sensitivity [175, 176]. In 2011, Cohen and colleagues found that changes in membrane potential could induce a detectable absorbance shift in the retinal chromophore of green-absorbing proteorhodopsin [151], opening up the possibility of using rhodopsins as voltage sensors and detectors. In their initial report, they used a proteorhodopsin mutant (D97N) lacking its proton-pumping capability, designated PROPS, to detect electrical spiking in *Escherichia coli* [151]. However, the probe failed to localize to the plasma membrane of mammalian cells [152]. Fortunately, other rhodopsins, prominently Archaerhodopsin 3 (Arch), a light-driven proton-pump from *Halorubrum sodomense* [117], do not suffer similar localization issues. In cultured hippocampal neurons, Arch robustly detected single electrically evoked AP with an optical SNR >10 and shows a sub-millisecond response time [152]. A non-pumping mutant of Arch (Arch-D95N) can significantly reduce the photocurrent generated by light irradiation and has a high degree of sensitivity (50% greater than Arch) to resolve single APs, albeit with a slower response (30–36 ms) [152].

A serious drawback that soon became apparent with rhodoposin-based GEVIs was their intrinsic low brightness (quantum yield of Arch is only 0.0009) [152]. Rational mutagenesis and directed evolution methods targeting residues that modulate the Schiff base charge have identified Arch mutants showing higher brightness and sensitivity, such as Arch-EEQ, Arch-EEN [153], and Arch-7 [177], although these Arch mutants are still two to three orders of magnitude dimmer than commonly used FP fluorophores (QY of EGFP is 0.6). Nevertheless, mechanistic insights into the voltage-sensitive fluorescence of Arch and other rhodopsins [175, 176, 178], combined with novel screening platforms [154], still hold much promise for the discovery of improved rhodopsin-based GEVIs. The recent engineering of QuarsArs [154], which are Arch variants with improved brightness (QuarsAr1 is 15-fold brighter than Arch), sensitivity (90% ΔF/F per 100 mV for QuarsAr2), and kinetics (0.05 ms and 1.2 ms for QuarsAr1 and QuarsAr2, respectively), exemplifies such endeavors. Interestingly, combined use of QuarsAr with a spectrally compatible optogenetic actuator, CheRiff, in a coexpression vector enables all-optical electrophysiology (Optopatch) in mammalian neurons—totally abolishing the need for conventional electrodes [154]. More recently, Flytzanis et al. engineered two Arch variants with enhanced radiance (Archers) and demonstrated their use in probing voltage dynamics in behaving *C. elegans* [156]. Notably, Archer1 has wavelength-specific dual functionality, as a voltage sensor under red light and as an inhibitory actuator under green light [156]. The development of Optopatch and Archer1 presages an exciting era of all-optical neurophysiology [154].

Alternative strategies to enhance the brightness of rhodopsin-derived GEVIs focused on FP-opsin fusions, whereby voltage induced absorbance changes of the opsin quench the emission of its brighter FP partner via electrochromic FRET (eFRET) [154]. As fluorescence change is usually detected in the FP channel, much less laser intensity is needed. Although FP-opsin fusions generally have slower response kinetics compared to standalone opsins, as observed in QuarsAr2-Citrine [154] and MacQ-mCitrine (Figure 3D) [155], the combined benefits of high voltage sensitivity of opsins and high brightness of FPs still enable these GEVIs to reliably detect single APs and sub-threshold voltage dynamics [155], thereby outperforming VSFPs, such as ArcLight. Furthermore, swapping FP spectral variants and optimizing the intervening FP-opsin linker are promising strategies toward further enhancing the performance of FP-opsin eFRET sensors [154]. Combined use of a fast response rhodopsin (*Acetabularia acetabulum* rhodopsin, Ace) and a bright FP (mNeonGreen) gave rise to Ace-mNeon, an ultra-fast GEVI that enable high-fidelity imaging of fast spike trains in live mice and flies [157].

## 4. Genetically Encoded Fluorescent Indicators of Synaptic Activity

Synaptic transmission is a signature event for neuronal information processing downstream of neuronal firing as it plays essential roles in information processing and memory formation [179]. During this process, APs approaching the synaptic bouton prompt presynaptic vesicles to exocytose and release neurotransmitters into the synaptic cleft, allowing neurons to communicate with each other via electrochemical signaling [180]. The development of indicators for synaptic activity lags far behind indicators for calcium and of membrane voltage [181]. Indicators of this class include those purposed for detecting neurotransmitter concentrations (such as glutamate) and pH changes during synaptic vesicle recycling (Table 3).

### 4.1. Genetically Encoded Glutamate Indicators

**Figure 4:**
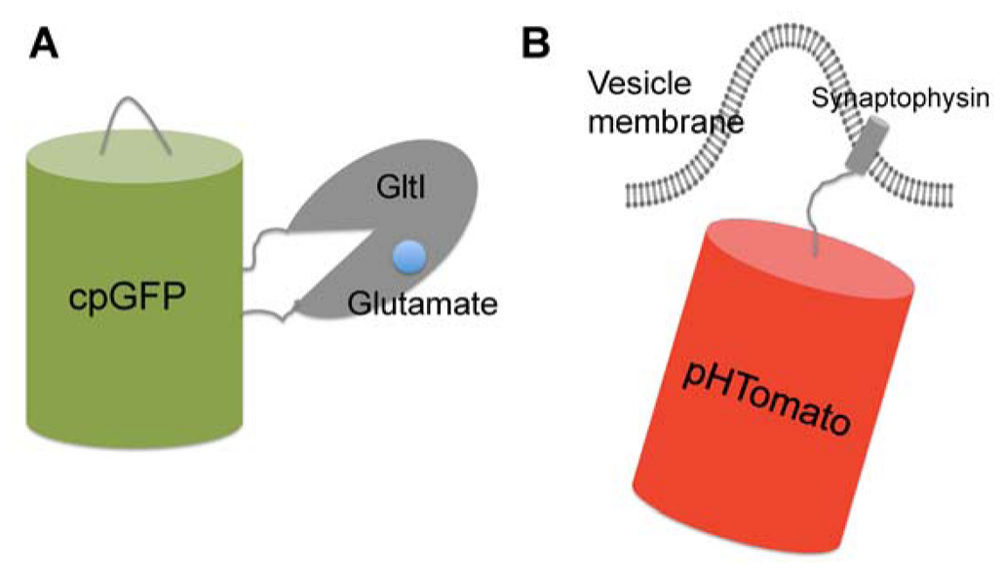
Schematic representation of genetically encoded indicators for synaptic activity (**A**) iGluSnFR is a genetically encoded single-FP-based glutamate indicator which contains a cpGFP inserted into the bacterial periplasmic glutamate binding domain (GlutI). Glutamate binding induced conformation change of Gltl results in deprotonation and fluorescence enhancement of cpGFP; (**B**) sypHTomato is a genetically encoded fluorescent reporter for synaptic vesicle recycling. It consists of a pH-sensitive red fluorescent protein (pHTomato) fused to the C-terminus of the vesicular protein domain Synaptophysin that localizes the probe to vesicle membrane. During membrane fusion, FP is switched from the low pH environment of vesicle to the neutral extracellular space, which leads to pH-dependent fluorescence changes. Subsequent vesicle recycling events reset the pH cycle.

Glutamate is a major excitatory neurotransmitter in the brain [190]. Postsynaptic glutamate release and extrasynaptic glutamate signaling (‘spillover’) give rise to many important neuronal processes, including synaptic crosstalk, learning, and memory [191, 192]. In 2005, Okumuto et al. reported the first fluorescent indicator protein for glutamate (FLIPE) by bracketing the bacterial periplasmic glutamate binding protein ybeJ (also known as “GltI”) [193] with CFP and Venus [182]. Glutamate binding triggers a conformational change within GltI, leading to a detectable FRET efficiency change. Tsien and colleagues later adopted a similar design and created a glutamate-sensing fluorescent reporter (GluSnFR) [183]. Both indicators showed glutamate-dependent FRET ratio changes in vitro*,* but their limited response amplitudes was insufficient for quantitation applications in neurons. Linker and binding affinity optimizations of GluSnFR gave rise to an enhanced probe, Super GluSnFR (44% ΔF/F), that was successfully used to quantitatively image synaptic glutamate spillover and reuptake in cultured hippocampal neurons with centisecond temporal and dendritic spine-sized spatial resolution [184]. In addition, an intensity-based, glutamate-sensing, fluorescent reporter (iGluSnFR) derived from cpEGFP insertion into GltI, was engineered with SNR and kinetics appropriate for in vivo imaging (Figure 4A) [185]. With a large dynamic range (4.5 ΔF/F) and fast response kinetics, iGluSnFR enables visualization of glutamate neurotransmission in intact neurological systems, including somata, dendrites, and dendritic spines in mouse retina, worms, zebrafish, and mice [185].

### 4.2. Genetically Encoded pH Indicators for Synaptic Vesicle Recycling

During the neurotransmitter-containing vesicles fusion to the presynaptic membrane upon AP propagation, the lumen of the vesicle, which is normally maintained at an acidic pH ~5.6, is exposed to the extracellular space (pH ~ 7.4). Subsequent vesicle recycling resets the acidity of the vesicle pH [194, 195]. Thus, tracking the vesicle pH changes associated with vesicle exocytosis and recycling events represent a plausible way to report on synaptic transmission. pH-sensitive variants of GFP are well suited to such applications. The first genetically encoded pH indicator tailored for this application is SynaptopHluorin, in which a pH sensitive GFP (pHluorin) was fused to the C-terminus of synaptobrevin/VAMP2 (vesicular associated membrane protein-2) [168]. The indicator was successfully localized to the inner surface of synaptic vesicles and faithfully reported local pH changes associated with transmissions at individual synaptic boutons. To explore the in vivo application of synaptopHluorin as a neurotransmitter indicator, Ng et al. targeted the probe to all three classes of neurons in the antennal lobe of *Drosophila* and performed functional imaging of olfactory circuits in response to natural odors [196]; Bozza et al. expressed the probe in mice mature olfactory sensory neurons, allowing them to monitor odorant-evoked activation of the sensory neurons in glomeruli of the olfactory bulb, and to reveal the spatial patterns of odorant-activated glomeruli [197]. Alternative targeting domains, such as synaptophysin [186] and vesicular glutamate transporter (vGlt1) [187], were used to better target pHluorin to the synaptic vesicle. Red-shifted variants of synaptopHluorin, including VGLUT1-mOrange2 [188] and sypHTomato (Figure 4B) [189], were also developed by switching pHluorin to pH-sensitive, red-shifted FP mutants. These color variants are spectrally compatible with GCaMP and permit concurrent measurements of calcium dynamics and synaptic vesicle recycling [188, 189].

## 5. Future Perspectives

As with electrophysiology, genetically encoded fluorescent probes offer a powerful approach to interrogate neuronal activities. While a plethora of fluorescent probes have been successfully developed and deployed to analyze neuronal-specific parameters, such as calcium, membrane voltage, neurotransmitter, and synaptic vesicle recycling, the currently available sensor toolbox, experimental methodologies, and optical modalities fall short of achieving the ultimate goal of proficiently investigating systems neuroscience, i.e., understanding how populations of nerve and glial cells form circuits underlying behaviors in awaken animals. The progress made in the past two decades with fluorescent probe development provides a strong foundation for future innovations aiming to realize the full potential of these optical probes for which research opportunities and challenges coexist and await further exploration.

First of all, the toolbox of genetically encoded fluorescent probes need to be further expanded to supplement currently widely used calcium and voltage indicators. Neuronal firing involves a cascade of signaling activities from which rich information are encoded and transmitted. While calcium and voltage play pivotal role in this process, other species, such as neurotransmitters, neuropeptides, and neuromodulators are also of paramount importance, but for which very few probes are available. In particular, indicators for many neurotransmitters, such as glutamate, γ-aminobutyric acid (GABA), histamine, serotonin, zinc ion [198] and dopamine remain attractive but hard-to-make [199]. Since voltage, calcium, and neurotransmitter changes represent functionally relevant, yet nonlinear, aspects of synaptic transmission [200], reporters for each class are of distinct value to study neuronal circuits. The development and in vivo application of the glutamate indicator iGluSnFR [185] have already showcased its indisputable potential in this area. In addition to creating new indicators for distinct species, further optimization of existing indicators will facilitate the recording of neuronal actives with higher SNRs and higher fidelity over the spatiotemporal scales most relevant to neurophysiological conditions. Importantly, both single-FP and FRET-based indicators have their own merits and limitations, and thus, should be selected with caution for specific applications. Single-FP based intensiometric biosensors typically have larger SNR than FRET-based biosensors, but are often sensitive to probe concentration and pH changes; ratiometric probes often suffer from high background signals due to the intrinsic inefficiency of the FRET process. Moreover, turn-off responses are less favorable than turn-on responses as photobleaching may complicate data interpretation. Furthermore, exploration of new sensing mechanisms such as FPX may provide ample opportunities for further innovation. In addition, indicators with red-shifted spectra (ideally near-infrared) are desirable for in vivo applications because they can potentially alleviate the light scattering and tissue penetration issues [201] encountered by commonly used green fluorescence indicators. Conceivably, red-shifted indicators can be used simultaneously with the reiteratively optimized GCaMP calcium indicators or blue light-activated channelrhodopsins [202, 203] to record and manipulate neuronal signals in multiplexing imaging experiments, as these indicators are spectrally orthogonal to each other. Parallel to the development of new indicators, the very nature of these fast and interconnected neuronal activities naturally calls for continuous optimizations of the brightness, photostability, response kinetics, and dynamic ranges of existing indicators, some of which have already found proof-of-concept demonstrations in vivo. In particular, the dynamic range and SNR of current GEVIs are far from optimal and necessitate major engineering efforts before their wide spread adoption.

The continuing advancement in the bioluminescence technology is also sparking a revolution in the development of bioluminescent sensors for brain imaging. On the one hand, caged luciferins are powerful tools to detect small and low abundant species such as ATP [204], copper-(I) [205], nitric oxide [206], and highly reactive oxygen species (hROS) [207]. In response to specific analytes, these caged luciferins were converted to better substrates for luciferases, resulting in enhanced bioluminescence signals. On the other hand, by swapping the donor FPs in existing FRET-based biosensors with luciferases, new chemiluminescent BRET biosensors were developed. In particular, the replacement of CFP in FRET sensors with NanoLuc resulted in BRET sensors with good sensitivity and response magnitude. For example, BTeam [208] modified from ATeam, LOTUS-V [209] modified from Mermaid2, and BLZinCh [210] modified from eZinCh-2 are NanoLuc-based BRET biosensors for ATP, membrane voltage, and Zn^2+^, respectively. We expect that the recently developed teLuc-cyOFP1 BRET pair may be utilized to further enhance these BRET-based biosensors [96]. As most FRET-based biosensors are modular, this approach could in principle be applicable to the development of BRET biosensors for various biomolecules or functions. Moreover, one may directly insert sensory domains into luciferases, such as NanoLuc and teLuc, to derive intensiometric, bioluminescent biosensors. Together, these efforts will create a versatile optical toolbox for deciphering the logics of neural activity in live, non-sedated animals.

Of similar importance to probe development is the advancement in optical instrumentation and imaging data processing. Recent progresses in multiphoton imaging [211–213], digital light sheet microscopy [214], aberration-corrected multifocus microscopy [215], and spatial light modulator microscopy [216] hold much potential for evolutionary modalities for brain imaging [217]. Accordingly, imaging acquisition and data processing methods [8, 22] need to be updated to accommodate larger and faster scales of information extraction from experiments.

Finally, optimization of the transduction methods to deliver these probes to specific cell or tissue types will be crucial to achieve spatial resolution in brain regions of interest. These include careful choice of promoters and vectors in viral packaging, and on-demand optimization of other transgenic techniques, such as in utero electroporation and stable transgenesis. Novel genome editing techniques, such as clustered regularly interspaced short palindromic repeats (CRISPR)-Cas9 [218] may also play pivotal roles in standardizing and streamlining the generation of transgenic rodent lines expressing various probes.

## Figures and Tables

**Table 1:** A list of genetically encoded Ca^2+^ indicators.

GECIs	Type a	Sensing Module	Reporting Module	Dynamic Range b	Selected Applications	Ref.
Cameleon-1	FRET	CaM-M13	BFP-GFP	70%	HeLa cells	[31]
YC2.1	FRET	CaM-M13	ECFP-EYFP	100%	HeLa cells; hippocampal neuron	[32]
YC3.6	FRET	CaM-M13	ECFP-cp173Venus	560%	HeLa cells; transgenic mice	[33]
YC6.1	FRET	CaM, CKKp	CFP-YFP	200%	HeLa cells; hippocampal neuron	[34]
D3cpV	FRET	CaM-M13	ECFP-cpVenus	510%	Hippocampal neurons	[35]

YC-Nano	FRET	CaM-M13	ECFP-cp173Venus	1450%	Layer 2/3 pyramidal neurons; zebrafish embryo	[36]

TN-L15	FRET	csTnC	CFP-Citrine	140%	HEK293 cells and primary hippocampal neurons	[37]

TN-XL	FRET	csTnC	ECFP-Citrine cp174	400%	Presynaptic motoneuron terminals of transgenic fruit flies	[38]

TN-XXL	FRET	csTnC	ECFP-Citrine Cp174	510%	Drosophila motor neuron boutons; mouse visual cortex	[39]

3xCFP–TnC–cpVenus	FRET	csTnC	3xCFP/cpVenus	1165%	PC12 Cells	[40]

Twitch-2B	FRET	tsTnC	cpVenus^CD^ and mCerulean3	>1000%	Mouse brain and T lymph nodes	[41]

BRAC	BRET	CaM-M13	Venus, RLuc8	60%	HeLa cells and plant leaves	[42]

Nano-lantern (Ca^2+^)	BRET	CaM-M13	Venus, split RLuc8	300%	HeLa cells and hippocampal neurons	[43]

Camgaroo-1	Single-FP	CaM	EYFP	700%	HeLa cells	[44]
Camgaroo-2	Single-FP	CaM	Citrine	700%	HeLa cells	[45]
Pericam	Single-FP	CaM, M13	cpEYFP	800%	HeLa cells	[46]
GCaMP1.3	Single-FP	CaM, M13	cpEGFP	450%	HEK-293 cells and mouse myotubes	[47]

GCaMP1.6	Single-FP	CaM, M13	cpEGFP	480%	Presynaptic boutons of the *Drosophila* larval neuromuscular junction	[48]

GCaMP2	Single-FP	CaM, M13	cpEGFP	390%	Beams of parallel fibers and granule cells of frontal cerebellar slices from transgenic mice	[49]

GCaMP3	Single-FP	CaM, M13	cpEGFP	1230%	Flies, worms and mice	[50]
GCaMP4.1	Single-FP	CaM, M13	cpEGFP	ND.	Xenopus laevis	[51]

GCaMP-HS	Single-FP	CaM, M13	cpEGFP	410%	Spinal motor neurons in transgenic zebrafish	[52]

GCaMP5	Single-FP	CaM, M13	cpEGFP	>940%	Cultured neurons and astrocytes, mouse retina; flies, worms, zebrafish and mice	[53]

Fast-GCaMP	Single-FP	CaM, M13	cpEGFP	>650%	Drosophila auditory neurons; superior cervical ganglion and neocortical pyramidal neurons	[54]

GCaMP6	Single-FP	CaM, M13	cpEGFP	>3600%	Cultured neurons; zebrafish, flies and mice	[55]

G-GECO1	Single-FP	CaM, M13	cpEGFP	2500%	HeLa cells; rat hippocampal neurons	[56]
R-GECO1	Single-FP	CaM, M13	cpmApple	1600%	HeLa cells; rat hippocampal neurons	[56]
B-GECO1	Single-FP	CaM, M13	cpEBFP	700%	HeLa cells	[56]
GEX-GECO1	Single-FP	CaM, M13	cpEGFP	2600%	HeLa cells	[56]
GEM-GECO1	Single-FP	CaM, M13	cpEGFP	11,000%	HeLa cells; transgenic *C. elegans*	[56]

CAR-GECO1	Single-FP	CaM, M13	cpmPulm	2700%	HeLa cells; mouse neocortical slice culture	[57]

R-GECO1.2	Single-FP	CaM, M13	cpmPulm	3300%	HeLa cells and INS-1 cells	[57]
O-GECO1	Single-FP	CaM, M13	cpmOrange	14,600%	HeLa cells	[57]

R-CaMP1.07	Single-FP	CaM, M13	cpmApple	2870%	HeLa cells and hippocampal pyramidal neurons	[58]

Y-GECO1	Single-FP	CaM, M13	cpmPapaya0.4	20,000%	HeLa cells and rat hippocampal neurons	[59]

REX-GECO1	Single-FP	CaM, M13	cpmApple	10,000%	Organotypic hippocampal slice cultures; albino tadpoles	[60]

GR-GECO	Single-FP	CaM, M13	cpmMapple145	450%	HeLa cells and rat hippocampal neurons	[61]

LAR-GECO	Single-FP	CaM, M13	cp146mApple	1000%	HeLa, HEK293, U2OS cells and rat hippocampal neurons	[62]

BCaMP1c	Single-FP	CaM, M13	cpBFP	200%	ND	[63]
CyCaMP1a	Single-FP	CaM, M13	cpCFP	260%	ND	[63]
YCaMP1b	Single-FP	CaM, M13	cpYFP	920%	HEK293 cells	[63]

RCaMP1h	Single-FP	CaM, M13	cp159mRuby	1050%	HEK293 cells, neurons; worms, fly larvae, and zebrafish	[63]

RA-CaM-B-M13-GA	FPX	CaM, M13	RA, B, GA	ND	HeLa cells	[64]

a FRET (Förster Resonance Energy Transfer) and single-FP (Fluorescent protein) types indicate ratiometric and intensometric response, respectively;

b Dynamic range here refers to the ratio of optical response in the fully activated indicator to the measured signal in the off state. Unless otherwise specified in the main text, the reported value refers to a positive relationship between Ca^2+^ and fluorescence intensity or ratio.

**Table 2:** A List of genetically encoded voltage indicators.

GEVIs	Sensing Module	Reporter Module	Dynamic Range (%) a	τ_on, fast_ (ms) b	τ_off, fast_ (ms) c	Selected Applications	Ref.
FlaSh	Shaker K^+^ channel	GFP	5.1	85 ± 10	160 ± 12	*Xenopus laevis* oocytes	[130]
SPARC	Rat μI skeletal muscle voltage-gated Na^+^ channel	GFP	0.5	<0.8	N.A.	*Xenopus laevis* oocytes	[131]
VSFP1	Kv potassium channel	CFP, YFP	1.8 ± 0.1	0.7	N.A.	HEK cells	[132]
VSFP2.1	CiVSD	Cerulean, Citrine	8.6	15	75	PC12 cells	[133]
VSFP2.3	CiVSD	Cerulean, Citrine	15.2 ± 0.2	3.0 ± 0.4	91.6 ± 4.2	PC12 cells	[134]
VSFP2.42	CiVSD	mCitrine, mKate2	12.46 ± 1.0	N.A.	N.A.	PC12 cells	[135]
CiVSD-Kv3.1 chimera (C5)	Ci-VSP-Kv3.1 VSD chimeras	mCerulean, mCitrine	14.8 ± 0.1	2.1	13.4	PC12 cells	[136]
VSFP-CR	CiVSD	Clover, mRuby2	12.7	5.4	90	Hippocampal neurons	[137]
VSF3.1	CiVSD	Cerulean	1.9	1.8 ± 0.3	N.A.	PC12 cells	[134]
VSFP3.1_mOrange2	CiVSD	mOrange2	2.9	3.8 ± 0.3	N.A.	PC12 cells and hippocampal neurons	[138]
VSFP-Butterfly1.2	CiVSD	mCitrine, mKate2	15.0 ± 0.7	1.0 ± 0.7	89.9 ± 5.2	Cortical neurons, barrel cortex and hippocampal slices	[139]
VSFP-Butterfly CY	Ci-VSP-Kv3.1 VSD chimeras	mCerulean, mCitrine	2.1 ± 0.2	14.6 ± 0.5	14.7 ± 0.2	HEK293 and PC12 cells; cortical neurons of living mice	[140]
VSFP-Butterfly-YR	Ci-VSP-Kv3.1 VSD chimeras	mCitrine, mKate2	2.3 ± 0.2	25.1 ± 0.9	12.7 ± 0.1	HEK293 and PC12 cells	[140]
Mermaid	CiVSD	mUKG, mKOκ	40	5–20	5–20	Rat cardiomyocytes and cortical neurons	[141]
Mermaid2	CiVSD	CFP, YFP	48.5	0.92	10.3	Hippocampal neurons and living mice	[142]
ArcLight Q239	CiVSD	Super ecliptic pHluorin A227D	35	9	17	HEK293 cells and hippocampal neurons	[143]
Chicken ArcLight-A173	Chicken VSD	Super ecliptic pHluorin A227D	9	4	9	HEK293 cells and cortical neurons	[144]
Bongwoori	CiVSD A154D/R217Q/R229I	Super ecliptic pHluorin A227D	~16	8	7	HEK293 cells and hippocampal neurons	[145]
FlicR1	CiVSD	cpmApple	6.6	3.0	2.8	HEK293 cells and hippocampal neurons and brain slices	[146]
Marina	ArcLight A389 A390 V442		31 d	29.2	15.6	HEK 293 cells and cortical neuronal cells	[147]
ASAP1	GgVSD	cpsfGFP-OPT	~18–29	2.1	2.0	HEK293A cells and hippocampal neurons	[148]
ASAP2s	ASAP1 R415Q		38.7	5.2	24	HEK293A cells, cardiomyocytes, *Drosophila* and organotypic slice cultures	[149]
ASAP2f	ASAP1 (A147S ΔA148)		~14–20	2.8	2.4	*Drosophila* visual system	[150]
PROPS	Proteorhodopsin		150	4.7	N.A.	*Escherichia coli*	[151]
Arch D95N	Arch-D95N		60	<1	<1	Rat hippocampal neurons	[152]
ArchEEQ	Arch-D95Q/D106E		60	~5–15	N.A.	Rat hippocampal neurons	[153]
ArchEEN	Arch-D95N/D106E		20	~5–15	N.A.	Rat hippocampal neurons	[153]
QuasAr1	Arch-P60S/T80S/D95H/D106H/F161V		32	0.05	0.07	Rat hippocampal neurons	[154]
QuasAr2	Arch-P60S/T80S/D95Q/D106H/F161V		90	1.2	1.0	Rat hippocampal neurons; hiPSC-derived neurons; organotypic brain slice	[154]
QuarsAr2-mOrange	QuarsAr2, mOrange		10	3.9	4.3	HEK293 cells and rat hippocampal neurons	[154]
QuarsAr2-mCitrine	QuarsAr2, mCitrine		13.1	3.1	4.8	HEK293 cells and rat hippocampal neurons	[154]
MacQ-mCitrine	*L. maculans* rhodopsin (Mac) D139Q, mCitrine		20	2.8	5.4	Cultured neurons; neocortical tissue slices; dendrites of Purkinje neurons in live mice	[155]
Archer1	Arch-D95E/T99C		85	N.A.	N.A.	Rat hippocampal neurons and sensory neurons in behaving *C. elegans*	[156]
Archer2	Arch-D95E/T99C/A225M		60	N.A.	N.A.	Rat hippocampal neurons	[156]
Ace-mNeon	*Acetabularia acetabulum* rhodopsinl (Ace), mNeonGreen		8.5–12	0.36–1.1	0.42–1.3	Neurons, awake mice and flies	[157]

a Dynamic range (%) here refers to the absolute value of fluorescence change (ΔF) normalized by the initial fluorescence (F_0_), expressed as % ΔF/F_0_, following 100 mV depolarization or repolarization steps. Values are steady-state or peak changes, as determined in the original reports. One has to be cautious when comparing these numbers, because experimental conditions could be different;

b,c The value here refers to one of the representative values measured in one cell type under a certain experimental condition specified in the original work, and may differ in other publications repeating the original work;

d Unlike other similar GEVIs (e.g., ArcLight, ASAP1, ASAP2s, ASAP2f, and Ace-mNeon) that have a “negative” response (i.e., decrease in fluorescence upon neuronal activation), Marina was engineered for an inverted response (i.e., increase in fluorescence upon neuronal activation).

**Table 3:** A List of genetically encoded indicators for synaptic activity.

Indicators	Type	Sensing Module	Reporting Module	Dynamic Range	Selected Applications	Ref.
FLIPE	FRET	GltI	ECFP, Venus	<5%	Rat hippocampal neurons and PC12 cells	[182]
GluSnFR	FRET	GltI	ECFP, Citrine	7.1%	Hippocampal neurons	[183]
SuperGluSnFR	FRET	GltI	ECFP, Citrine	44%	Hippocampal neurons	[184]
iGluSnFR	Single-FP	GltI	cpEGFP	450%	Cultured neurons; retina, worms, zebrafish and mice	[185]
SynaptopHluorin	Single-FP	pHluorin	VAMP2-pHluorin	8–20%	Hippocampal neurons; RBL-2H3 cells	[168]
sypHy	Single-FP	pHluorin	synaptophysin-pHluorin	N.A.	Hippocampal neurons	[186]
vGpH	Single-FP	pHluorin	vGlut1-pHluorin	N.A.	Hippocampal boutons	[187]
VGLUT1-mOrange2	Single-FP	mOrange2	VGLUT1-mOrange2	16%	Hippocampal boutons	[188]
sypHTomato	Single-FP	pHTomato	Synaptophysin-pHTomato	5–25%	CA3-CA1 hippocampal neurons	[189]
